# IDO-1 impairs antitumor immunity of natural killer cells in triple-negative breast cancer via up-regulation of HLA-G

**DOI:** 10.1007/s12282-023-01522-w

**Published:** 2023-11-19

**Authors:** Rui Jing, Shukun Bai, Peipei Zhang, Hao Ren, Lintao Jia, Weimiao Li, Guoxu Zheng

**Affiliations:** 1 College of Life Sciences, Northwest University, Xi’an, 710069 China; 2 State Key Laboratory of Holistic Integrative Management of Gastrointestinal Cancers and Department of Biochemistry and Molecular Biology, Fourth Military Medical University, Xi’an, 710032 China; 3 Department of Oncology, The Second Affiliated Hospital of Xi’an Jiaotong University, 157 Xiwu Road, Xi’an, 710004 China; 4 State Key Laboratory of Holistic Integrative Management of Gastrointestinal Cancers and Department of Immunology, Fourth Military Medical University, 169 Changle West Road, Xi’an, 710032 China; 5 Department of Urology, Tangdu Hospital, Fourth Military Medical University, Xi’an, 710038 China

**Keywords:** TNBC, IDO-1, NK cell, HLA-G, Immunotherapy

## Abstract

**Background:**

Triple-negative breast cancers (TNBC) are highly aggressive malignancies with poor prognosis. As an essential enzyme in the tryptophan–kynurenine metabolic pathway, indoleamine 2,3 dioxygenase-1 (IDO-1) has been reported to facilitate immune escape of various tumors. However, the mechanism underlying the immunosuppressive role of IDO-1 in TNBC remains largely uncharacterized.

**Methods:**

We examined the IDO-1 expression in 93 clinical TNBC tissues and paired adjacent normal tissues, and analyzed the regulation role of environmental cytokines like IFN-γ in IDO-1 expression. The effect of IDO-1 expression in TNBC cells on the function of NK cells were then evaluated and the underlying mechanisms were exploited.

**Results:**

IDO-1 expressed in 50 of 93 (54.1%) TNBC patients. TNBC patients with high IDO-1 expression tended to have more infiltrated immune cells including NK cells, which are less active than patients with low IDO-1 expression. NK cells could produce IFN-γ, which induced IDO-1 expression in TNBC cells, whereas IDO-1 impaired the cytotoxicity of co-cultured NK cells by upregulation of HLA-G. Blockade of HLA-G improved the antitumor activity of NK cells to TNBC in vivo.

**Conclusion:**

TNBC cells induce dysfunction of NK cells through an IFN-γ/IDO-1/HLA-G pathway, which provide novel insights into the mechanisms of TNBC progression and demonstrate the applicability of IDO-1 and HLA-G targeting in the treatment of TNBC.

## Introduction

Breast cancer (BC) is divided into four subtypes: Luminal A, Luminal B, HER2-positive, and triple-negative breast cancer (TNBC). Among them, TNBC is the most aggressive subtype with the worst prognosis [1]. Traditional chemotherapy and adjuvant chemotherapy have limited efficacy in clinical practice, and the systemic toxicity of traditional therapeutics and drug resistance have brought challenges to the treatment of TNBC. Denkert et al. found that there are more tumor-infiltrating lymphocytes (TILs) in TNBC tissues. With the increase in TILs infiltration in TNBC, the overall survival of patients has also been prolonged, which suggest immunotherapy as an innovative strategy for treating TNBC [2]. The use of immune checkpoint inhibitors (ICIs) such as PD-1 antibodies in the immunotherapy of TNBC patients has been extensively studied, but the clinical efficiency is so far unsatisfactory [3, 4].

Indoleamine 2,3 dioxygenase-1 (IDO-1) is an essential enzyme in the tryptophan–kynurenine metabolic pathway, and can be induced by IFN-γ, a cytokine produced by different myeloid lineage cells [5]. In the physiological conditions, IDO-1 is secreted by maternal placental trophoblast cells to inhibit T cell activity through regulating tryptophan metabolism, which is important in maintaining tolerance at the fetal-maternal interface [6]. In tumor microenvironment, IDO-1 is highly expressed by dendritic cells, tumor-associated macrophages, myeloid-derived suppressor cells, and cancer-associated fibroblasts, which are all associated with immunosuppressive effects [7, 8]. Mechanistically, IDO-1 can trigger cell cycle arrest in the G1 phase or autophagy in T cells by activation of amino acid-sensitive kinase GCN2 and inhibition of the mTOR signaling pathway via tryptophan–kynurenine metabolic pathway in tumor microenvironment. In addition, the binding of tryptophan metabolites to the aromatic hydrocarbon receptors can counteract signals that activate T cells, leading to immune escape of tumor cells [9, 10]. However, it has not been clearly elucidated if IDO-1 can regulate natural killer (NK) cell activity in the microenvironment of solid tumors like TNBC.

Human leukocyte antigen-G (HLA-G) is a non-classical major histocompatibility complex (MHC) class I molecule. During pregnancy, HLA-G expressed by maternal placental trophoblast cells can induce spiral artery remodeling and bind to immune cells, which leads to fetal-maternal immune tolerance [11]. Recent studies have demonstrated that when HLA-G is highly expressed, tumor cells are more likely to escape from the immune surveillance [12, 13]. Clinically, HLA-G has become a preoperative diagnostic marker and prognostic indicator [14]. In a prior study, we discovered that HLA-G binds to the NK cell receptor KIR2DL4, desensitizing HER2-positive breast cancer cells to trastuzumab. Trastuzumab responsiveness for HER2-positive breast cancer can be improved by blocking the HLA-G/KIR2DL4 signaling pathway [15]. Considering the involvement of both IDO-1 and HLA-G in feto-maternal tolerance, it is worth exploring whether they cooperate to induce immunosuppression in the context of tumor progression. In this study, we found that IDO-1, upon induction by tumor environmental cytokines like IFN-γ, can suppress the tumoricidal capacity of NK cells by up-regulating HLA-G. These findings provide new insights into the mechanisms underlying the immunosuppressive role of IDO-1, and have implications for the feasibility of IDO-1 and HLA-G targeting in clinical therapy of TNBC.

## Materials and methods

### Patients

This study was conducted in accordance with the ethical guidelines stipulated in the 2013 Declaration of Helsinki. The Ethics Committee of the Second Affiliated Hospital of Xi’an Jiaotong University (No.2023192) approved the histochemical analysis of samples from the patients. Written informed consent for the sample collection and use was obtained from all patients. All authors accessed, reviewed, and approved the data and contents of the manuscript. Cancer tissue and paired para-cancerous tissue of 93 TNBC patients from 2017 to 2018 were collected by the department of oncology of the Second Affiliated Hospital of Xi’an Jiaotong University to investigate the expression of IDO-1 in TNBC patients. The diagnosis of TNBC was histologically confirmed in 93 patients who were at least 18 years old as described previously [16]. The inclusion requirements included a minimum 3 month survival rate, adequate bone marrow, hepatic, and renal function, and signed informed permission. Incomplete case information, post-operative death from complications, and the presence of other cancers were all considered exclusion criteria.

### Cell culture and reagents

HEK293T cells and human TNBC cell lines (MDA-MB-231 and HCC-1937) were obtained from American Type Culture Collection (Manassas, USA). All cell lines were cultured in Dulbecco’s modified Eagle’s medium (Thermo Fisher, Waltham, USA) supplemented with 10% heat-inactivated fetal bovine serum (Thermo Fisher, Waltham, USA) at 37 °C in a humidified 5% CO_2_ incubator.

### Plasmid construction and lentivirus infection

Short hairpin RNAs (shRNAs) targeting IDO-1 and its flanking control sequence were cloned in pLKO.1-EGFP-puro vector. The target sequence was as follows: shIDO-1#1: 5ʹ-CCATCTGCAAATCGTGACTAA-3ʹ, shIDO-1#2: 5ʹ-CGTAAGGTCTTGCCAAGAAAT-3ʹ, shIDO-1#3: 5ʹ-CGCTGTTGGAAATAGCTTCTT-3ʹ, shNC: 5ʹ-GGTTCTCCGAACGTGTCACGT-3ʹ. The qualified plasmids were then transfected using Lipofectamine 2000 (Invitrogen, Carlsbad, USA) into HEK293T cells for packaging. At 48 or 72 h following transfection, virus-containing supernatant was harvested and centrifuged to remove cell debris, and then stored at – 80 ℃ until use. MDA-MB-231 cells in the logarithmic growth phase were washed with phosphate-buffered saline (PBS), adjusted to a cell density of 5 × 10^5^ cells/mL, and re-inoculated into a 6-well dish. The recombinant lentiviruses were used to infect the cells for 48 h, followed by selection of infected cells using puromycin.

### Western blot

Cells were lysed in lysis buffer (50 mM NaCl, 50 mM EDTA, and 1% Triton X-100) supplemented with a protease inhibitor cocktail (Roche, Basel, Switzerland). Cell lysates were separated by SDS-polyacrylamide gel electrophoresis and transferred to nitrocellulose membranes. Membranes were then blocked with 5% nonfat milk diluted in PBS for 2 h at room temperature and incubated with the following primary antibodies: anti-IDO-1 (Cat.No.86630S, Cell Signaling Technology, Boston, USA); anti-HLA-G (Cat.No.66447-1-Ig, Proteintech, Wuhan, China), and anti-GAPDH (Cat.No.60004-1-Ig, Proteintech, Wuhan, China). Then, membranes were washed with PBS containing 0.05% Tween and incubated with secondary antibodies conjugated with HRP for 1 h at room temperature. The bands were developed using a chemiluminescence reagent (Thermo Fisher, Waltham, USA).

### Flow cytometry

Primary NK cells were analyzed via FCM using antibodies as follows: APC-labeled anti-human CD56 antibody (Cat.No.362503, Biolegend, California, USA) and FITC labeled anti human CD3-antibody (Cat.No.300405, Biolegend, California, USA). Briefly, 5 × 10^5^ cells were washed and resuspended in PBS buffer containing 2% bovine serum albumin and 0.2% sodium azide and incubated with either directly labeled antibody or unconjugated antibody (followed by directly labeled secondary antibody) for 30 min at 4 °C. After incubation, the cells were washed three times and analyzed using FCM (Becton, Dickinson and Company, New Jersey, USA).

### In vitro NK cell expansion

All experiment protocols are approved by the Ethics Committee of the Fourth Military Medical University. NK cells were isolated from the peripheral blood of healthy adult donors. Written informed consent was obtained from all participants. PBMCs were isolated by Ficoll density gradient centrifugation, and NK cells were purified by negative magnetic selection using an NK Cell Isolation Kit (Cat.No.130-092-657, Miltenyi, Teterow, Germany) following the manufacturer’s instructions. NK cells were expanded in the RPMI 1640 medium (Thermo Fisher, Waltham, USA) supplemented with 10% FBS (Thermo Fisher, Waltham, USA) and 10 ng/ml anti-CD3 antibody (Cat.No.GTX79905, GeneTex, California, USA), and 15 ng/ml IL-2 (Cat.No.200-02, PeproTech, New Jersey, USA) and 50 ng/ml IL-15 (Cat.No.200-15, PeproTech, New Jersey, USA) were added every other day. Cells from expansion days 14–21 with purity > 90% were used for further experiments.

### siRNA synthesis and transfection

The small interfering RNAs (siRNAs) targeting IDO-1 were synthesized by GenePharma (Shanghai, China). The siRNA was transfected into TNBC cells using Lipofectamine 2000 (Invitrogen, Carlsbad, USA) according to the manufacturer’s instructions. The siRNA sequence used is as follows: siIDO-1 #1, 5′-CAAAGUAAUUCCUACUGUATT-3′; siIDO-1 #2, 5′-GUAUGAAGGGUUCUGGGAATT-3′; and negative control, 5′-UUCUCCGAACGUGUCACGUTT-3′. Cells were collected for analysis 48 h after transfection with indicated siRNA.

### Quantitative real-time PCR

Total RNA was extracted using TRIzol reagent (Thermo Fisher, Waltham, USA) following the manufacturer’s instruction. RNA was reverse transcribed using the Prime-Script RT Reagent Kit (TaKaRa, Kusatsu, Japan). Quantitative PCR was conducted using a CFX96TM Real-Time PCR system (Bio-Rad, Hercules, USA) with SYBR Green Reagents (TaKaRa, Kusatsu, Japan). Gene expression levels were normalized to that of GAPDH. The primers used for real-time PCR were as follows: IDO-1-F:5ʹ-GCCAGCTTCGAGAAAGAGTTG-3ʹ, IDO-1-R:5ʹ-ATCCCAGAACTAGACGTGCAA-3ʹ; HLA-G-F: 5ʹ-CGTCCTGGGTCTGGTCCT-3ʹ, HLA-G-R:5ʹ-GTGGCTCCACAGATACCTG-3ʹ; GAPDH-F:5ʹ-AGGTCCACCACTGACACGTT-3ʹ, GAPDH-R:5ʹ-GCCTCAAGATCATCAGCAAT-3ʹ.

### ELISA

Human IL-2 (Cat.No.1110202, Dakewe, Shenzhen, China), human TNF-α (Cat.No.1117202, Dakewe, Shenzhen, China) and human IFN-γ (Cat.No.1110002, Dakewe, Shenzhen, China) in the tissue supernatants or cell supernatants were quantified using commercially available ELISA Kits following the manufacturer’s instructions. Briefly, the supernatants were added to an ELISA Kit plate that was precoated with a specific antibody. A biotinylated secondary antibody was then added and the plate was incubated at room temperature for 2 h. The color development catalyzed by HRP was terminated with 2.5 mol sulfuric acid, and the absorption was measured at 450 nm. The protein concentration was normalized to the relative absorbance rate of the respective standard and expressed as the mean ± SD.

### CCK8 assays

NK cell activity and the proliferation of IDO-1 knockdown TNBC cells or IDO-1 inhibitors (1-L-MT, Cat.No.447439, Sigma-Aldrich, Shanghai, China) treated TNBC cells were determined by CCK8 (Yeasen, Shanghai, China) according to the manufacturer’s instructions. Briefly, indicated cells in 96-well plates were incubated in 100 μl CCK-8 working solution at 37 °C for 2 h. The OD value was then measured by microplate reader (Thermo Fisher, Waltham, USA), at a wavelength of 450 nm. The killing rate of NK cells were estimated using the formula: [1 − (OD value of effector-target cell)/OD value of target cell] × 100%.

### Histochemical analysis

Paraffin-embedded tissue sections were subjected to IHC staining as described previously with minor modifications [17]. Briefly, the slides were deparaffinized in xylene and rehydrated in a graded alcohol series, and the endogenous peroxidase activity was blocked with 3% H_2_O_2_. Nonspecific binding sites were also blocked using pre-immune rabbit serum before incubation of the sections overnight in a humidity chamber at 4 °C with the following primary antibodies: anti-IDO-1 (Cat. No. ab156787, Abcam, Cambridge, UK). Slides were then washed three times with PBS, followed by incubation with a biotinylated secondary antibody for 30 min at room temperature. Signal was visualized by incubation with 3,3ʹ-diaminobenzidine chromogens for 2–3 min. The IHC results were quantified according to the staining intensity and extent as previously described [18]. Briefly, the staining intensity was scored from 0 to 3 points (0 for no staining, 1 for weak immunoreactivity, 2 for moderate immunoreactivity and 3 for strong immunoreactivity). The score for positive percentage was determined as follows: 0 for all negative cells, 1 for < 25% positive cells, 2 for 25–50% positive cells, 3 for 50–75% positive cells, and 4 for more than 75% positive cells. The final score was obtained by multiplying the intensity score and positive proportion score, and ranged from 0 to 12. For immunofluorescent staining of tumor tissues, the sections were deparaffinized and blocked as aforementioned, and were incubated at 4 °C overnight with primary antibodies against IDO-1 (Cat. No. ab156787, Abcam, Cambridge, UK), CD69 (Cat. No. PA5-102562, Invitrogen, Carlsbad, USA) and NKG2D (Cat. No. PA5-97904, Invitrogen, Carlsbad, USA). Subsequently, incubation with goat anti-mouse IgG H&L (Alexa Fluor^®^ 488) (Cat. No. ab150113, Abcam, Cambridge, UK) and goat anti-rabbit IgG H&L (Alexa Fluor^®^ 594) (Cat. No. ab150080, Abcam, Cambridge, UK) was performed for 1 h at 37 °C in the dark. Nuclei were counterstained with 4ʹ,6-diamidino-2-phenylindole. Fluorescent images were acquired by a confocal laser-scanning microscope (Ti2-E-A1, Nikon, Tokyo, Japan).

### Analysis of in vivo tumor growth

Animal experiments were approved by and conducted in full compliance with the regulations of the Institutional Animal Care and the Ethics Committee of the Fourth Military Medical University (No. 20220830 and KY20224215). Nude mice (6 weeks old, 18 ± 0.39 g weight, female) were purchased from Hunan Slater Jingda Experimental Animal Co., Ltd. All mice were fed autoclaved food and water. The right groin of mice was injected with 5 × 10^6^ control cells or IDO-1 knockdown MDA-MB-231 cells for the development of TNBC xenografts. Mice were randomly assigned into three groups receiving treatment with PBS, NK cells alone, or NK cells combined with an HLA-G-blocking antibody (1 mg/kg, Cat. No. MAB11223, Abnova, Taiwan, China) (*n* = 6 in each group). When tumor volume reached 200 mm^3^ (defined as day 0), NK cells and the antibody were administered via tail vein on days 0, 3 and 6. Tumor diameter was measured using a Vernier caliper every 5 days from day 5 until day 23. In order to analyze the anti-tumor efficiency of NK cells and HLA-G blocking antibody, mice were sacrificed on day 23. Tumor size was estimated using the formula: tumor volume = length × width^2^/2, where the length and width represented the longest and shortest tumor diameters.

### Statistics

SPSS 20.0 software was used for data processing and GraphPad Prism 8.0 for data analysis and graphing. Using the *t* test (unpaired), the data from the two groups were compared. For three groups and more than three groups, one-way ANOVA was utilized. Data were provided as mean ± standard deviation, and *P < *0.05 denoted a significantly difference in the outcomes.

## Results

### IDO-1 is highly expressed in TNBC tissue and correlated with immune cell infiltration

**Fig. 1:**
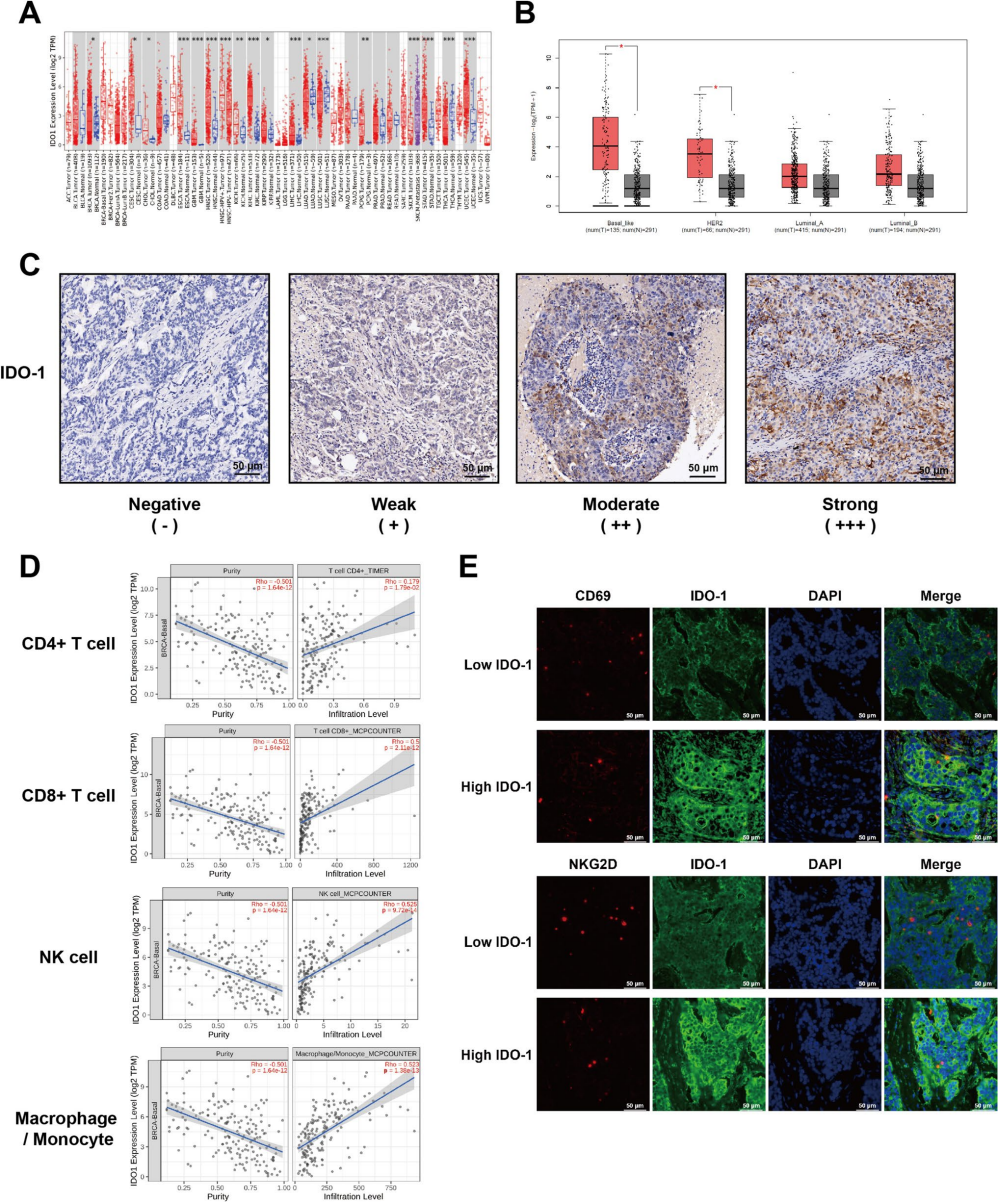
IDO-1 is highly expressed in triple-negative breast cancer. **A** The expression of IDO-1 in pan-cancer in the TIMER 2.0 database. **B** The expression of IDO-1 in breast cancers from the GEPIA dataset (basal-like, cancer tissue (*n* = 135); HER2, cancer tissue (*n* = 66); Luminal A, cancer tissue (*n* = 415); Luminal B, cancer tissue (*n* = 194)). Cancerous tissue marked “tumor” (red box) was compared to adjacent tissue marked “normal” (grey box). **C** IHC analysis of IDO-1 in human TNBC tissue specimens was performed and representative images with distinct IDO-1 levels are shown. Scale bar, 50 µm. **D** Correlation analysis for IDO-1 and immune cell infiltration in TNBC tissues. **E** Representative immunofluorescent staining of IDO-1, CD69, NKG2D in TNBC tissues of individual patients. Scale bar, 50 µm

We employed the TIMER2.0 database and GEPIA database to assess IDO-1 expression in TNBC tissues. We found that breast cancer tissues significantly expressed IDO-1 compared to healthy breast tissues, especially in TNBC subtype (Fig. 1A, B). IHC staining was also performed on 93 TNBC tumor samples and paired normal tissues. Results showed that 50 of 93 (54.1%) TNBC tissues have positive IDO-1 expression in cytoplasm of parenchymal cells of the tumor (Fig. 1C). The relationship between immune cell infiltration and IDO-1 expression was then examined, which revealed infiltration of more immune cells like NK cells, T cells and macrophages in TNBC tissues with a higher IDO-1 expression (Fig. 1D). To evaluate the activation status of the NK cells in TNBC patients, we performed immunofluorescence staining for the activated lymphocyte marker CD69 and the NK cell activating receptor NKG2D, and found that NK cells from tumor tissues with higher IDO-1 expression are less activated than those from tumors expressing lower IDO-1 (Fig. 1E). Thus, IDO-1 is abundantly expressed in TNBC, and the levels of IDO-1 are associated negatively with the activation of tumor-infiltrating NK cells.

### IDO-1 can be induced in TNBC cells by tumor environmental or NK cell-derived IFN-γ

**Fig. 2:**
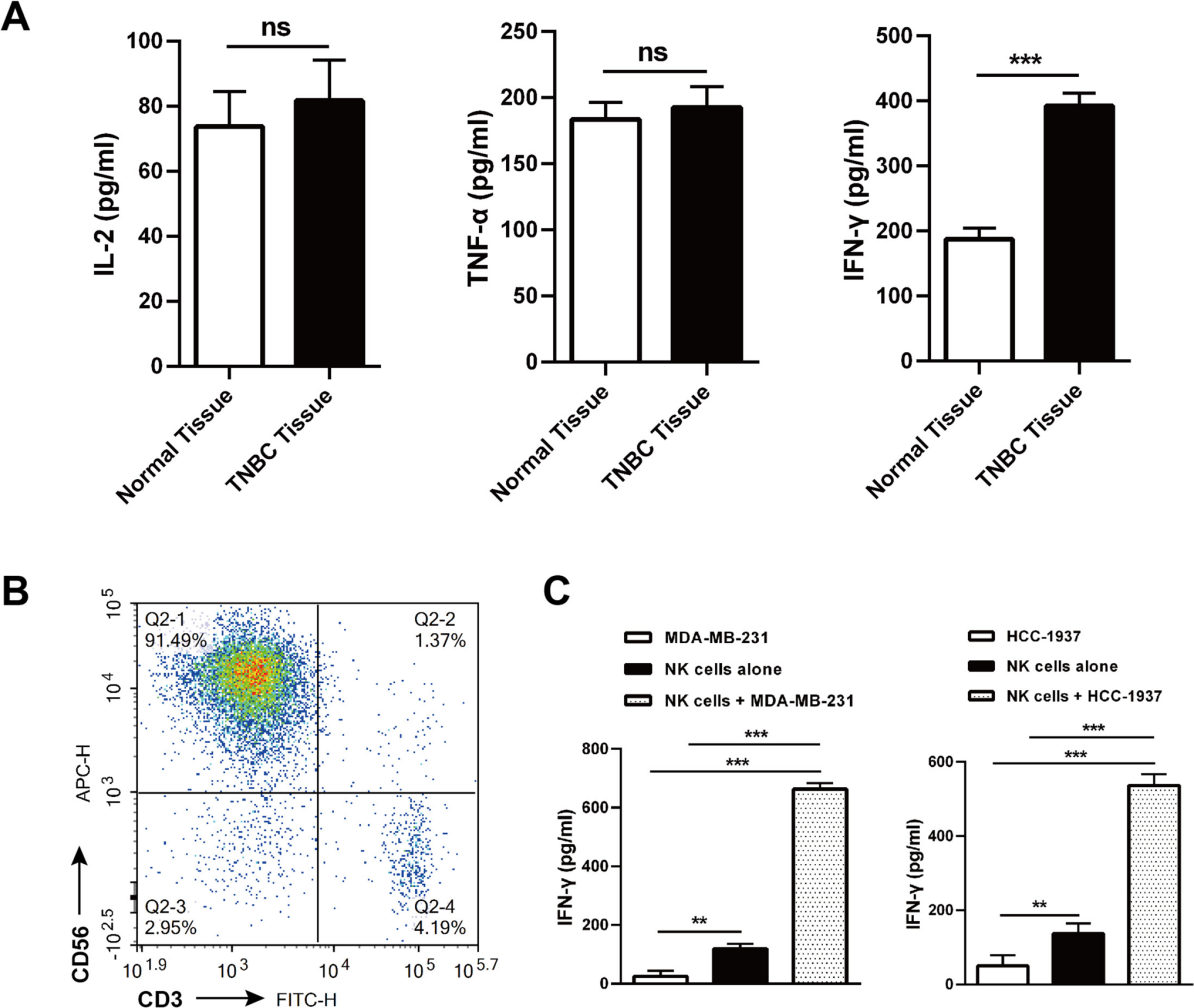
IFN-γ is produced by NK cells coexisting with TNBC cells. **A** Clinical TNBC and adjacent normal tissue samples were collected and subjected to ELISA for levels of indicated cytokines (*n* = 6). **B**, **C** NK cells were prepared from human PBMCs (**B**), and were cultured alone or cocultured with TNBC cells at a ratio of 5:1 for 48 h. The supernatants of cells were then harvested and subjected to ELISA (**C**). ns, non-significant; ^∗∗^*P* < 0.01, ^∗∗∗^*P* < 0.001

**Fig. 3:**
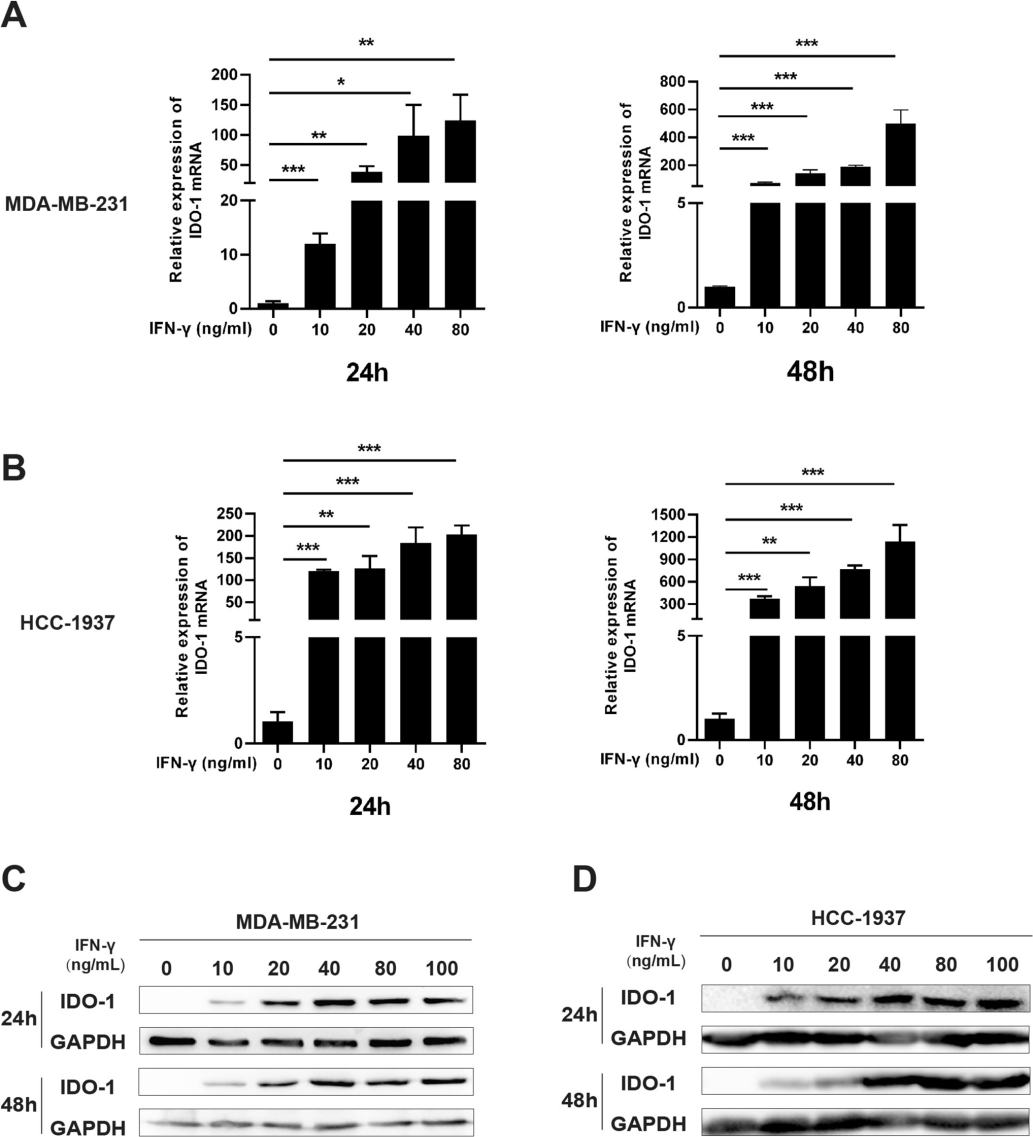
IFN-γ induces IDO-1 expression in TNBC cells. **A**, **B** MDA-MB-231 cells (**A**) or HCC-1937 cells (**B**) were cultured in the presence of indicated doses of IFN-γ for 24 or 48 h, and were subjected to qRT-PCR assays. **C**, **D** Cells were cultured as described in (**A**) and (**B**), and cell lysates were prepared for Western blot analysis. ^∗^*P* < 0.05, ^∗∗^*P* < 0.01, ^∗∗∗^*P* < 0.001

The microenvironment of solid tumors is featured by the enrichment of multiple immune-suppressive cytokines [19]. Consistently, we found that the levels of IFN-γ, not IL-2 and TNF-α, were remarkably higher in clinical TNBC than the adjacent normal tissues (Fig. 2A). We next prepared NK cells (CD3^−^CD56^+^) from human PBMCs by magnetic bead sorting (Fig. 2B), and found that when co-cultured with TNBC cells, these cells produce abundant IFN-γ, a cytokine known to license the transcription of *Ido-1* (Fig. 2C) [20]. The human *Ido-1* promoter contains two interferon-stimulated response elements (ISREs) that are required for IFN-γ-induced full transcription of the human IDO-1 gene [20]. To determine whether IDO-1 can be activated by IFN-γ in TNBC cells, we stimulated TNBC cells with different doses of IFN-γ for 24 and 48 h. Western blot and qRT-PCR analyses indicated that IDO-1 expression was detectable at 24 and 48 h after IFN-γ induction and it exhibited a definitive dose–effect relationship in the IFN-γ concentration range of 0–100 ng/mL (Fig. 3A–D). In the subsequent study, we used 80 ng/mL as the working concentration of IFN-γ to induce IDO-1 expression in TNBC cells. These data suggest that IDO-1 can be upregulated in TNBC cells by IFN-γ derived from lymphocytes including NK cells.

### IDO-1 expression can inhibit the cytotoxicity of NK cells to TNBC cells

**Fig. 4:**
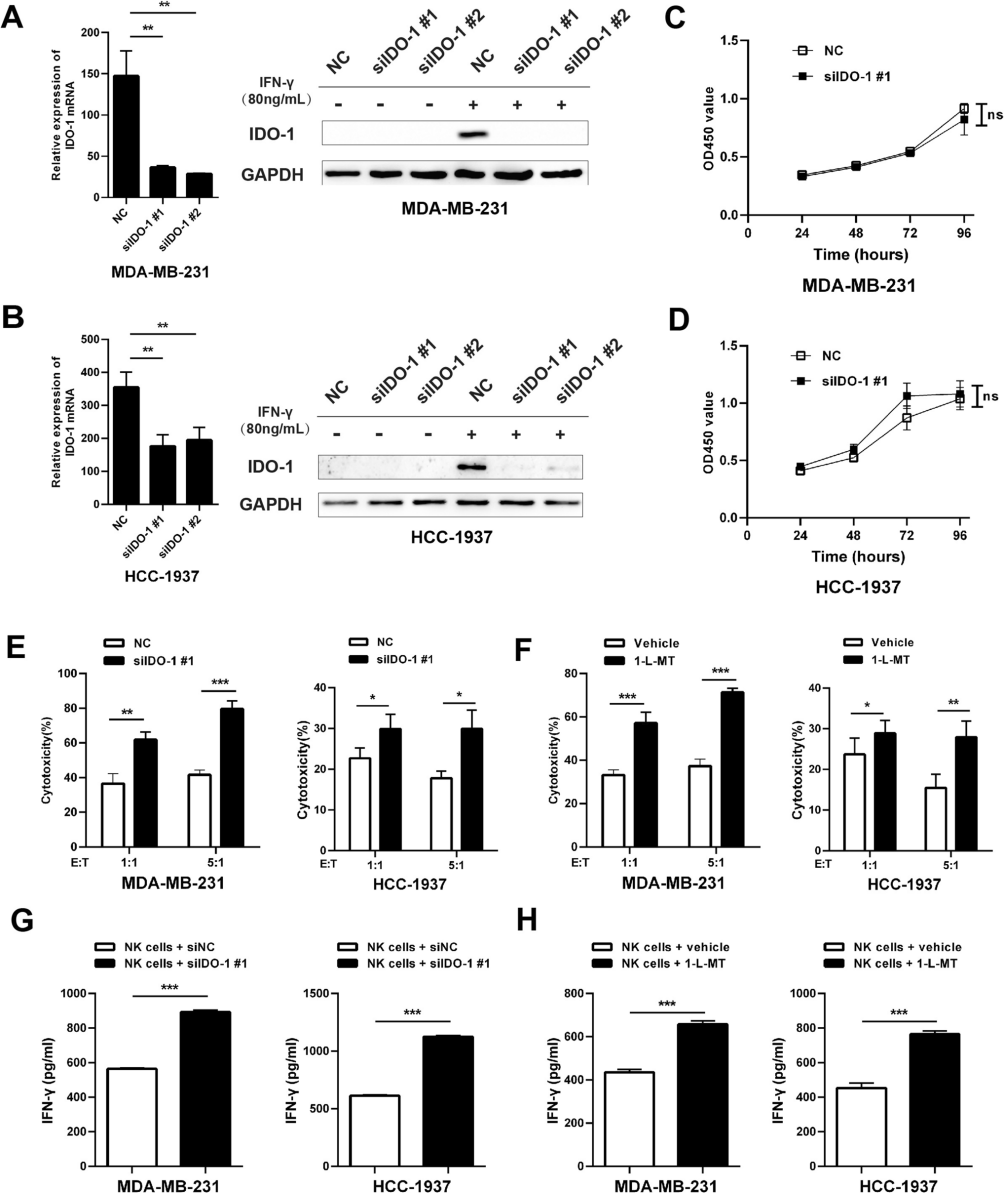
IDO-1 knockdown in TNBC cells improved the cytotoxicity of co-cultured NK cells. **A**, **B** TNBC Cells were transfected with indicated siRNAs and treated with or without IFN-γ for 72 h. The expression of IDO-1 was detected by qRT-PCR (left) and Western blot (right) analyses. **C**, **D** CCK8 assay was carried out to detect the survival and proliferation of control and IDO-1 knockdown TNBC cells. **E** NK cells were co-cultured with control or IDO-1 knockdown TNBC cells in the indicated ratios for 6 h, and the percentages of lyzed cells were measured. **F** TNBC cells were incubated with vehicle or 500 μM 1-methyl-L-tryptophan (1-L-MT) for 24 h, then, NK cells were co-cultured with TNBC cells in the indicated ratios for 6 h, and the percentages of lyzed cells were measured. **G** NK cells were co-cultured with control and IDO-1 knockdown TNBC cells in indicated ratios for 48 h (E:T = 5:1), and IFN-γ production was measured by ELISA. (H) TNBC cells were incubated with vehicle or 500 μM 1-methyl-L-tryptophan (1-L-MT) for 24 h, then, NK cells were co-cultured with TNBC cells in indicated ratios for 48 h (E:T = 5:1), and IFN-γ production was measured by ELISA. ns, non-significant; ^∗^*P* < 0.05, ^∗∗^*P* < 0.01, ^∗∗∗^*P* < 0.001

We next examine the function of IDO-1 in TNBC cells and its immune-suppressive role in tumorigenesis. TNBC cells were transfected with siRNAs targeting IDO-1 and treated with IFN-γ, and the knockdown of IFN-γ-induced IDO-1 was validated in both the mRNA and the protein levels (Fig. 4A, B). CCK8 assay showed that the knockdown of IDO-1 has no impact on the growth of TNBC cells (Fig. 4C, D). To determine how IDO-1 expression in TNBC cells affects cytotoxicity of NK cells, we co-cultured them with the IDO-1 knockdown TNBC cells or IDO-1 inhibitors treated TNBC cells at different ratios. CCK8 assay revealed that IDO-1 silencing or inhibition in TNBC cells could improve the cytolytic activity of co-cultured NK cells (Fig. 4E, F). Concomitantly, IDO-1 silencing or inhibition in TNBC cells can significantly increase IFN-γ production of NK cells (Fig. 4G, H). Therefore, high IDO-1 level in TNBC cells contributes to a compromised tumoricidal capability of local NK cells.

### IDO-1 impairs cytotoxicity of NK cells by up-regulating HLA-G in TNBC cells

**Fig. 5:**
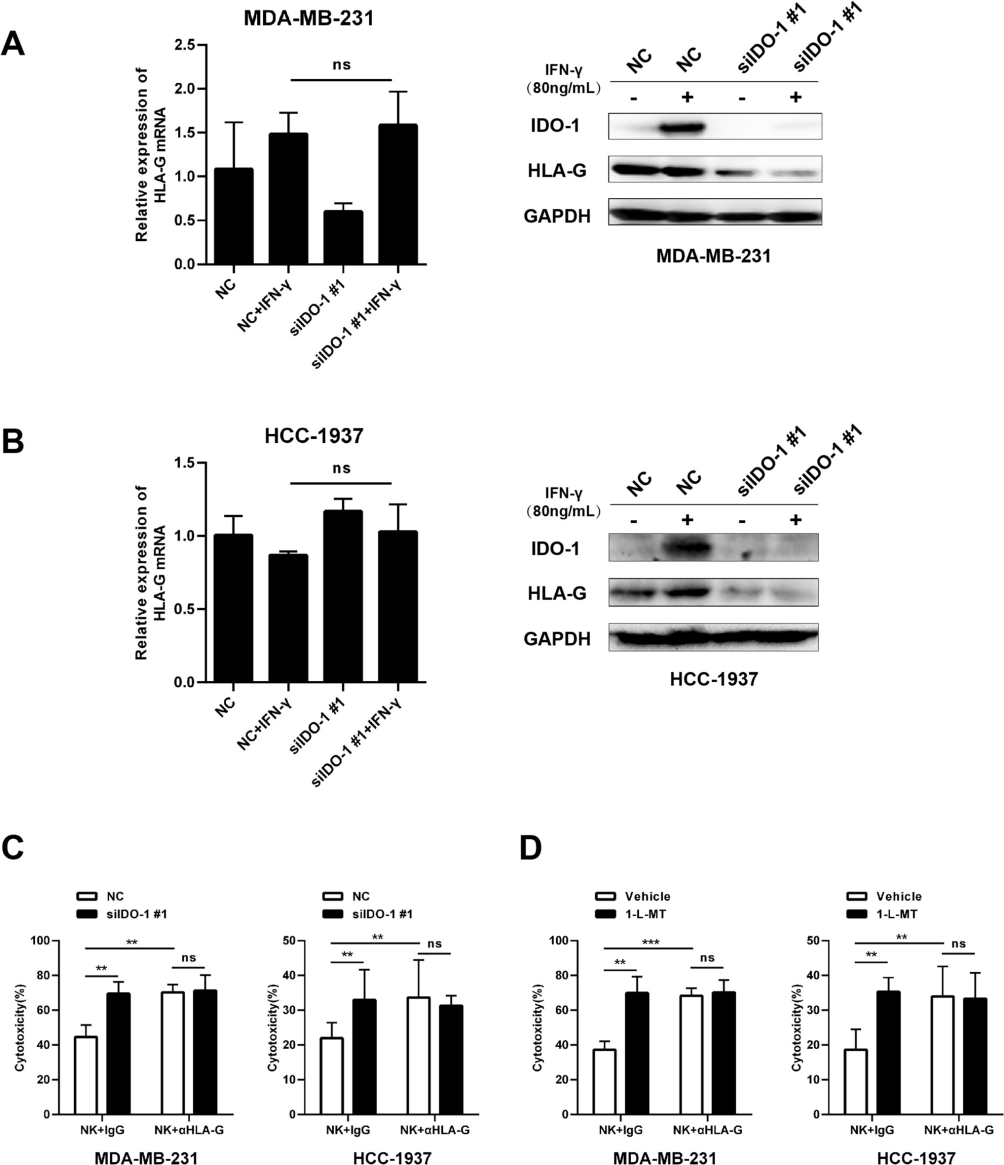
IDO-1 inhibits the cytotoxicity of NK cells by up-regulating HLA-G. **A**, **B** TNBC cells were transfected with indicated siRNAs and treated with or without IFN-γ for 72 h. The expression of indicated proteins was detected by qRT-PCR (left) and Western blot (right) analyses. **C** NK cells were co-cultured with control or IDO-1 knockdown TNBC cells in media supplemented with or without HLA-G blocking antibody (10 μg/mL) for 6 h (E:T = 5:1), and the percentages of lyzed cells were measured. **D** TNBC cells were incubated with vehicle or 500 μM 1-methyl-L-tryptophan (1-L-MT) for 24 h, then, NK cells were co-cultured with indicated TNBC cells in media supplemented with or without HLA-G blocking antibody (10 μg/mL) for 6 h (E:T = 5:1), and the percentages of lyzed cells were measured. ns, non-significant; ^∗∗^*P* < 0.01

The activity of NK cells is fine-tuned by a class of surface receptors and their cognate ligands expressed on tissue parenchymal cells, as exemplified by HLA-G/KIR2DL4 that delivers inhibitory signal to NK cells [15]. We then examined the expression of HLA-G after knockdown of IDO-1 in TNBC cells. As a result, we observed that IDO-1 silencing can reduce the level of HLA-G protein, although the mRNA level was not affected by knockdown of IDO-1 (Fig. 5A, B). While IDO-1 knockdown or inhibition and HLA-G blockade comparably enhanced cytolysis by NK cells, the HLA-G-blocking antibody failed to significantly improve the cytotoxicity of NK cells when co-cultured with IDO-1 knockdown TNBC cells or IDO-1 inhibitors treated TNBC cells (Fig. 5C, D). These data suggest that IDO-1 in TNBC cells attenuates the antitumor capacity of NK cells at least partially through HLA-G/KIR2DL4-mediated inhibition of NK cell activation.

### IDO-1/HLA-G can inhibit the antitumor activity of NK cells in vivo

**Fig. 6:**
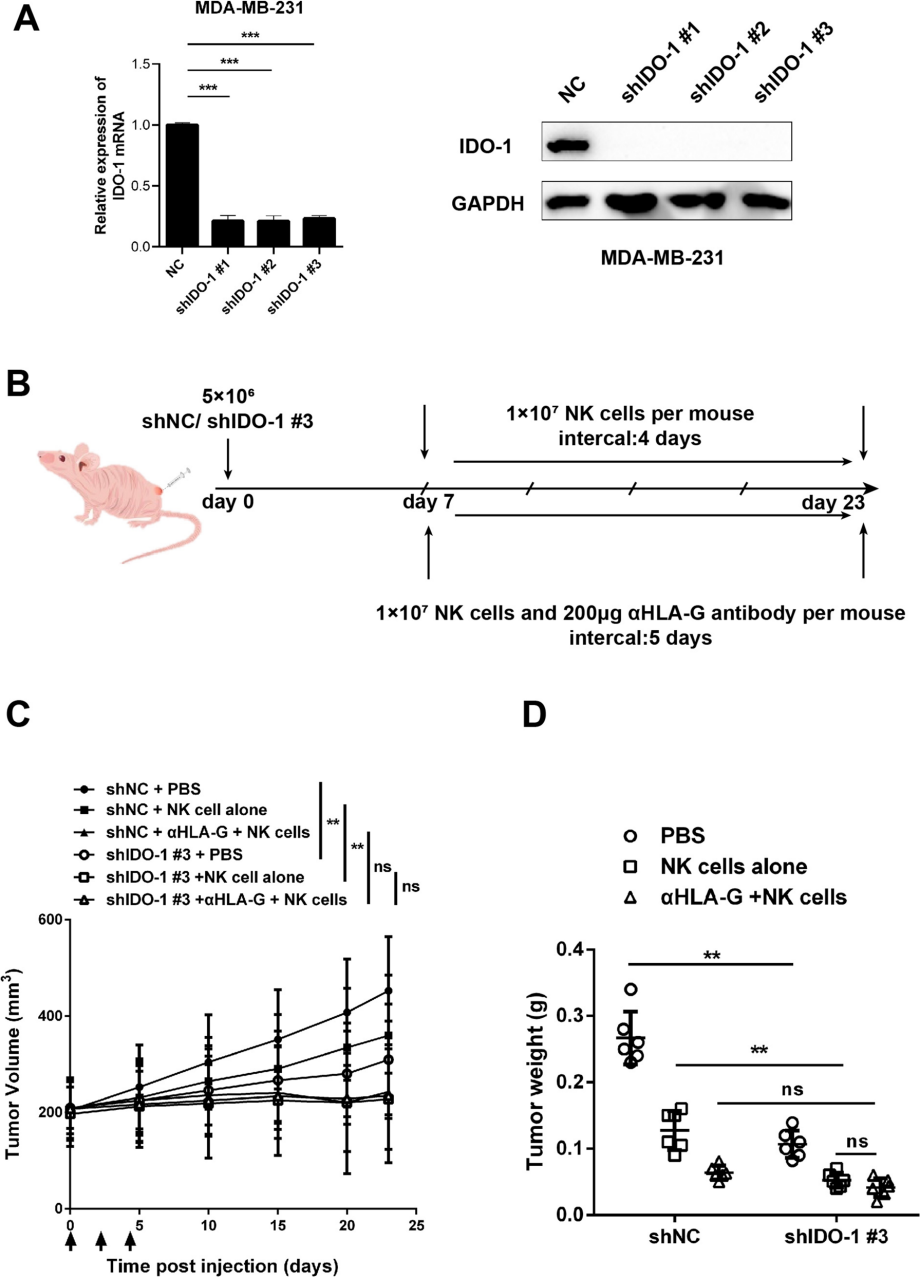
IDO-1 silencing and HLA-G blockade comparably promote repression of xenograft TNBC by infused NK cells. **A** Cells were infected with lentiviruses stably expressing the indicated shRNA, and were subjected to qRT-PCR and Western blot analyses. **B**–**D** A xenograft TNBC model was established, followed by infusion of NK cells for treatment (**B**). Mice were sacrificed and the tumors were isolated on day 23. The volume (**C**) and weight (**D**) of tumors were measured and plotted (*n* = 6). ns, non-significant; ^∗∗^*P* < 0.01, ^∗∗∗^*P* < 0.001

We established TNBC cells stably expressing the IDO-1-targeted short hairpin RNA (shRNA). As a result, sustained IDO-1 silencing was achieved in these cells (Fig. 6A). Then, we established a xenograft tumor model using control and IDO-1 knockdown MDA-MB-231 cells, followed by treatment via adoptive transfer of NK cells and/or injection with the HLA-G antibody (Fig. 6B). We found that infusion of NK cells resulted in a remarkable repression of tumor growth (Fig. 6C, D). However, although HLA-G neutralization further improved the therapeutic efficacy of NK cells on tumors derived from parental MDA-MB-231 cells, it displayed a modest effect on tumors developed from IDO-1 knockdown MDA-MB-231 cells (Fig. 6C, D). Thus, IDO-1 impedes the in vivo suppression of TNBC by NK cells mainly through upregulation of HLA-G on neoplastic cells.

## Discussion

The heterogeneity and lack of biomarkers are considered to be the main causes of treatment resistance, recurrence, and clinical treatment failure of TNBC. Although the use of immune checkpoint inhibitors can significantly prolong the survival of patients, their clinical application has been limited by the low therapeutic response and frequently occurring drug resistance, which are ascribed largely to the immunosuppressive microenvironment of solid tumors. Therefore, understanding the molecular machinery that induces the dysfunction of immune cells will provide new ideas for improving the effect of tumor immunotherapy. Some scholars have studied the clinicopathological features of early PD-L1-positive TNBC patients, and detected much higher levels of IDO-1 than those of PD-L1, suggesting a critical role of IDO-1 in the low response of patients to anti-PD-L1 therapy. Thus, IDO-1 has emerged as a crucial therapeutic target in the attempt to remodel the immuno-suppressive tumor microenvironment [21, 22]. Recent study showed that the Epacadostat, a highly selective IDO-1 inhibitor, in combination with pembrolizumab had encouraging antitumor activity in multiple advanced solid tumors, which indicated that IDO-1 might be a novel immune checkpoint [23]. In the present study, we established that TNBC cells exhibited a widely high expression of IDO-1 through preliminary analysis of the existing databases and subsequent immunohistochemistry analysis. In addition, TNBCs expressing high IDO-1 are featured by extensive immune cell infiltration and the inhibitory immune microenvironment. The above results suggest that IDO-1 may affect the prognosis of patients mainly by inducing the dysfunction of immune cells or educating them to facilitate tumor development.

Natural killer (NK) cells are a key subset of cells that play a fundamental role in immune surveillance against carcinogenesis as well as in the antitumor efficacy of therapeutic antibodies [24]. In addition to preventing the onset and progression of breast cancer, NK cells also aid in limiting the systemic spread of cancer stem cells. The overall survival rate of breast cancer patients was reported to correlate positively with the quantity of infiltrating NK cells in the tumor, while the metastasis of tumor draining lymph nodes was adversely associated with the homing NK cells [25]. The effector functions of NK cells are determined by integrated signals across the array of stimulatory/inhibitory receptors and the corresponding ligands on target cells. We previously found that breast cancer cells express HLA-G, which inhibits the cytotoxicity of NK cells by binding to the receptor KIR2DL4. Accumulating studies have demonstrated that oncometabolites generated by metabolic reprogramming are essential regulators of the immunosuppressive cancer microenvironment. In particular, Chiesa et al. found that kynurenine reduced the cytotoxicity of NK cell by inhibiting NK cell-activating receptors NKp46 and NKG2D [26]. We established here that high IDO-1 expression in TNBC cells upregulates HLA-G in the protein level, which contributes to compromised cytotoxicity and impaired antitumor capability of NK cells. However, the detailed mechanisms underlying IDO-1-mediated HLA-G upregulation remain to be deciphered, e.g. the metabolite(s) involved in the above pathway, or whether the increase in HLA-G is due to improved protein synthesis or impeded protein degradation. In addition, further studies are needed to investigate whether these regulatory events also play a role in maternal immunotolerance. Nonetheless, our findings provide a novel link between metabolic reprogramming and immune escape of cancer, and hold out promise for identification of new biomarkers for TNBC that could be targeted in combination with immunotherapy.

## Data Availability

The data that support the findings of this study are available on request from the corresponding author upon reasonable request.
